# Scanning Acoustic Microscopy (SAM): A Robust Method for Defect Detection during the Manufacturing Process of Ultrasound Probes for Medical Imaging

**DOI:** 10.3390/s19224868

**Published:** 2019-11-08

**Authors:** Francesco Bertocci, Andrea Grandoni, Tatjana Djuric-Rissner

**Affiliations:** 1 R&D Global Transducer Technology, Esaote spa, Via di Caciolle, 15, 50127 Firenze, Italy; andrea.grandoni@esaote.com; 2 PVA TePla Analytical Systems GmbH, 73463 Westhausen, Germany; tatjana.djuric-rissner@pvatepla.com

**Keywords:** quality improvement, industrial applications, capability enhancement, manufacturing process, scanning acoustic microscopy (SAM), non-destructive testing (NDT), damage detection and visualization, internal defects, failure analysis

## Abstract

The main aim of this paper is to provide the feasibility of non-destructive testing (NDT) method, such as scanning acoustic microscopy (SAM), for damage detection in ultrasound (US) probes for medical imaging during the manufacturing process. In a highly competitive and demanding electronics and biomedical market, reliable non-destructive methods for quality control and failure analysis of electronic components within multi-layered structures are strongly required. Any robust non-destructive method should be capable of dealing with the complexity of miniaturized assemblies, such as the acoustic stack of ultrasonic transducers. In this work, the application of SAM in an industrial scenario was studied for 24 samples of a phased array probe, in order to investigate potential internal integrity, to detect damages, and to assess the compliance of high-demanding quality requirements. Delamination, non-homogeneous layers with micron-thickness, and entrapped air bubbles (blisters) in the bulk of US probe acoustic stacks were detected and studied. Analysis of 2D images and defects visualization by means of ultrasound-based NDT method were compared with electroacoustic characterization (also following as pulse-echo test) of the US probe through an ad-hoc measurement system. SAM becomes very useful for defect detection in multilayered structures with a thickness of some microns by assuring low time-consuming (a limit for other NDT techniques) and quantitative analyses based on measurements. The study provides a tangible contribution and identifies an advantage for manufacturers of ultrasound probes that are oriented toward continuous improvement devoted to the process capability, product quality, and in-process inspection.

## 1. Introduction

In order to secure reliable manufacturing procedures, the effective and selective elimination of units with poor quality containing internal defects (i.e., delamination voids, cracks, or air bubble entrapment) plays a crucial role during the production of US probes for medical imaging, with the goal of reducing costs and improving the process capability. Therefore, an efficient non-destructive test method can be useful for finding and localizing such imperfections in the acoustic stack. There are many NDT methods [1] for failure analysis of damage and for defect detection in microelectronic packages and in composite materials, including, but not limiting to: Optical microscopy [2], infrared thermography (IRT) [3,4], X-ray computed tomography (XCT) [5], and scanning acoustic microscopy (SAM) [6,7]. Compared to scanning electron microscopy (SEM) [8] and transmission electron microscopy (TEM) [9,10], non-destructive techniques do not require time-consuming sample preparation with the further benefit of not damaging the unit under test. In fact, in most cases, i.e., for safety-relevant assemblies in aerospace [11], rolling stock [12], and automotive applications [13], it is mandatory to study failures and other damages through a nondestructive technique. These techniques become very efficient and effective for the quality and safety assessment of products.

Among different non-destructive methods, analysis by means of infrared thermography is influenced by ambient factors, i.e., moisture, temperature. X-ray scans depend strongly on the thickness and density of the sample under testing. The principal disadvantage consists in the low resolution of small defects in the bulk material. X-ray-based techniques are not effective in the case of multilayered structures that are composed of high Pb contents, such as the active layer of US probes based on piezoelectric material, i.e., PZT (lead zirconate titanate) or PMN-PT (lead magnesium niobate-lead titanate). In fact, the penetration degree of X-rays inside this type of advanced materials is limited. The authors tested the effectiveness of high-resolution 3D X-ray microscopy by means of SKYSCAN 1272 (14,450 × 14,450 pixels)—BRUKER, but the phase contrast and resolution did not distinguish the virtual slices through the samples and, therefore, the defects.

Optical microscopy is useful for a fast visual inspection, but it is strongly limited to a surface investigation.

Scanning acoustic microscopy is a non-destructive technique used since the early 1980s that is able to provide imaging of the internal structure of thick-film circuits [14] and multilayer ceramic capacitors [15] by the inspection of defects and delamination occurring during the manufacturing process. Even though it is a complex technique, requiring specific skill and experience for operation and interpretation of the results, it still provides some advantages over other available NDT technologies, which makes it an excellent choice in some applications. The possibility to view the internal structure of every sample in a non-destructive manner and to analyze the potential effects of the failure root causes makes SAM an important tool in a wide range of production processes. Defect inspection proved that SAM has high potential in microelectronics packaging [1,16,17] by detecting delamination [18], voids [19], and micro-cracks [20]. High confidence levels of failure localization and the reliability assessment in MEMS sensors [21] by means of scanning acoustic microscopy have been proved. Moreover, defect visualization in 3D packaging within the potential automatization of SAM for a fast and cost efficient failure inspection was reported in [22,23]. During the stages of the manufacturing process of complex and multi-layered structures, delamination can generate failures. These can be avoided by means of reliable material analysis, as determined for pins delamination of plastic-encapsulated microcircuits [24]. Furthermore, SAM is very useful for the quality and reliability improvement of aerospace products by detecting and eliminating faulty components in the early stages of the manufacturing process [25]. Experimental determination of the resolution and sensitivity of various transducers with frequencies up to 230 MHz has been conducted in systematic studies using well-defined calibration blocks, showing good agreement with the predicted resolution by diffraction theory [26,27,28].

Nevertheless, there are a lack of studies on damage detection in multi-layered structures composed of silicone, piezoelectric material, and epoxy resins, with an overall thickness in the range 300–700 µm. The authors present the feasibility of a scanning acoustic microscopy able to detect and identify internal defects in US probe acoustic stacks for medical imaging during the manufacturing process. The SAM investigation on a multi-layer structure of a US probe is a challenge. In fact, in a multilayered structure, with a decrease of the thicknesses of around some micro-meters, it becomes increasingly difficult to separate and distinguish echo-signals coming from different layers due to the overlapping of multiple reflections. Furthermore, the highest achievable resolution of SAM is around 1 µm by using high frequency (e.g., 1 GHz) in order to increase the lateral resolution [29,30], but the drawback is the limitation of the penetration depth around some microns. In fact, the attenuation obeys a frequency power law [31,32], thus when using GHz-SAM, only the volume within a few micro-meters below the sample’s surface can be analyzed.

The aim of this study is twofold. Firstly, this study aims to detect the presence of delamination, non-homogeneous layers, and air voids at micron thicknesses in the bulk of ultrasonic transducers through SAM. Secondly, it aims to predict the quality and faulty units in the middle of the manufacturing process with the purpose of improving the production yield and decreasing the cost of the waste material. Furthermore, the study is focused not only on the inspection and timing capability for each unit under detection but also on the resolution and penetration depth that is able to assess the integrity of US probe acoustic stacks. The ad-hoc setup of the scanning acoustic microscopy and the proper preparation of 24 samples are implemented and discussed. The quantitative analyses by means of SAM are compared with the destructive analysis, i.e., scanning electron microscopy, and with the electroacoustic characterization of the complete acoustic stacks. A comparison between the SAM measurements and pulse-echo test allows for an identification of the root cause occurring during the manufacturing process of US probes for medical imaging.

The paper is organized as follows. In Section 2, the basics of scanning acoustic microscopy are described. Section 3 introduces the technical problem on the manufacturing process for ultrasound probes for medical imaging. The experimental setup of the SAM, the preparation of the samples under testing, and the ad-hoc measurement system for the electroacoustic characterization of the products will be discussed in Section 4. In Section 5, the results of the tests and the NDT detection efficiency are presented. The discussion follows in Section 6. The conclusions close the paper in Section 7.

## 2. Principles of Scanning Acoustic Microscopy (SAM)

The most important part of a SAM device is the transducer. The latter sends an ultrasonic signal (TX) at a small point on the target object [1]. The frequency of the ultrasonic signal generated by the transducer is typically in the range 15–300 MHz up to GHz (in the case of high detailed resolution but limited penetration depth). In fact, the resolution and inspection depth of SAM measurements depends not only on the acoustic frequency but also on the transducer’s focal length, material properties, and complexity of the structure of the specimen under testing, in addition to the demanded information [29].

**Figure 1:**
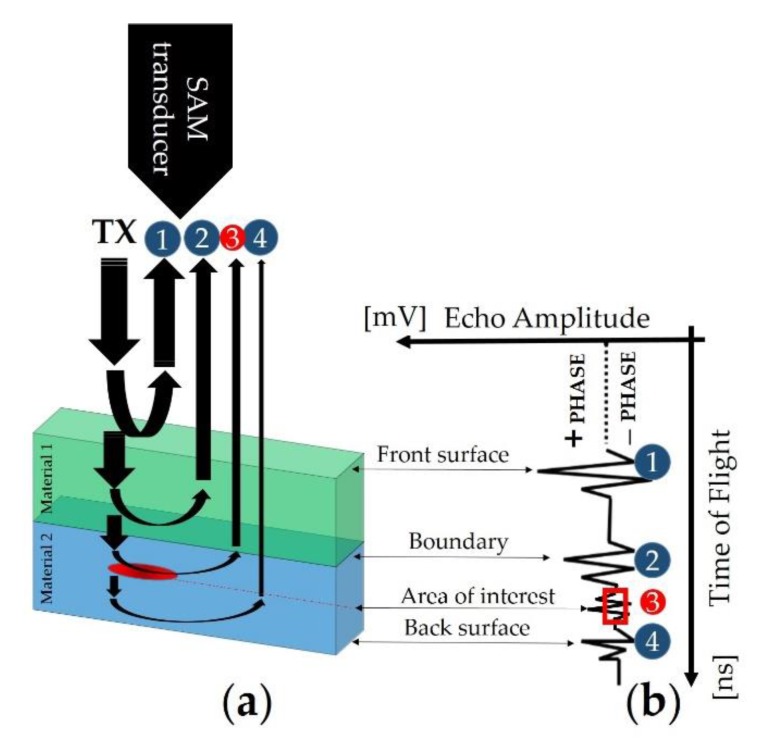
A-scan through the thickness of the sample under testing (target object).

Fractions of the incident acoustic energy by TX are back-reflected when there is a change in the acoustic impedance, Z, (1) between the interfaces of internal materials (Figure 1a) under inspection:

(1) $$Z_{i}=\;\rho_{i}\cdot v_{i}.$$

The index *i* is the material type, and *ρ* and *υ* are the density and the sound velocity of the material, respectively [33]. The exact portion of the back-reflected amplitude can by calculated by the reflection coefficient, *R*, equal to (2):

(2) $$R=\frac{Z_{2}-Z_{1}}{Z_{2}+Z_{1}}.$$

The higher the impedance mismatch at the interface, the higher the intensity of the reflected signal (more brightness in the 2D image), which is measured by the echo amplitude. In the case of an interface with air (Z = 0), total reflection of the ultrasonic wave occurs; therefore, SAM is highly sensitive to any entrapped air in the sample under testing.

Besides measuring the intensity of the reflected sound wave, the time needed for the detection of the back-reflected wave is also captured and displayed in the so-called A-scan (Figure 1b). Then, information about the depth (location), *d*, of a potential defect in the material bulk can be found by:

(3) $$d\;=\;\frac{ToF\cdot c}{2},$$

in which time of flight (*ToF*) is measurable in the A-scan and *c* is the sound speed of the material. The division by 2 is explained by the back and forth trip of the sound wave from and to the SAM transducer. In the generic sample structure of Figure 1a, the sequence of the reflected waveforms (Figure 1b) is composed of the signal #1 that is commonly referred to as the front surface, i.e., first interface. The same behavior is connected for signal #2 due to the interface between material 1 and material 2. The waveform #3 would be considered the area of interest. The red box (data gate) indicates the depth of information, which is positioned over this signal or group of signals for evaluation. The signal #4 is referred to the back surface that indicates the bottom of the sample. Then, the echo signals are analyzed and processed by considering the location of particles, voids, air bubbles, delamination, or cracks through Equation (3).

**Figure 2:**
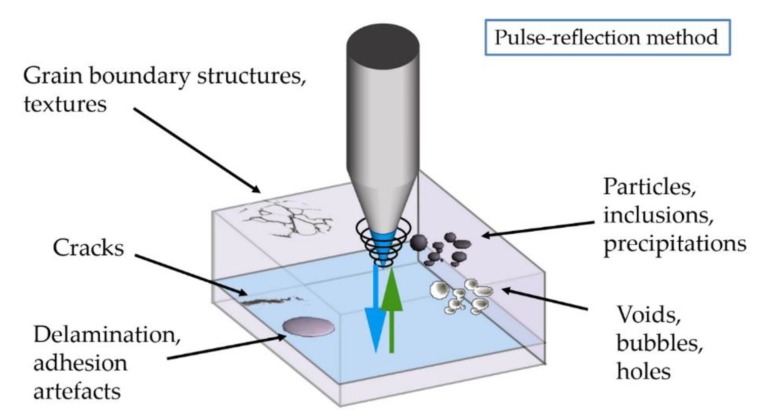
Defect detection and sample interaction (image courtesy of PVA TePla Analytical Systems).

Therefore, 2D or 3D-dimensional images of the internal structure become available by means of the pulse-reflection method, in which the impedance mismatch between two materials leads to a reflection of the ultrasonic beam (Figure 2). Phase inversion of the reflected signal can allow for discrimination of the delamination (acoustic impedance almost zero) from inclusions and particles, but not from air bubbles, which show same impedance behavior as delamination. In the case of the application of SAM to the US probe acoustic stack, phase inversion can be affected from the multiple reflections occurring due to the multilayered structure, so it is tough to attribute the phase inversion to delamination or bubbles located in deeper interfaces.

**Figure 3:**
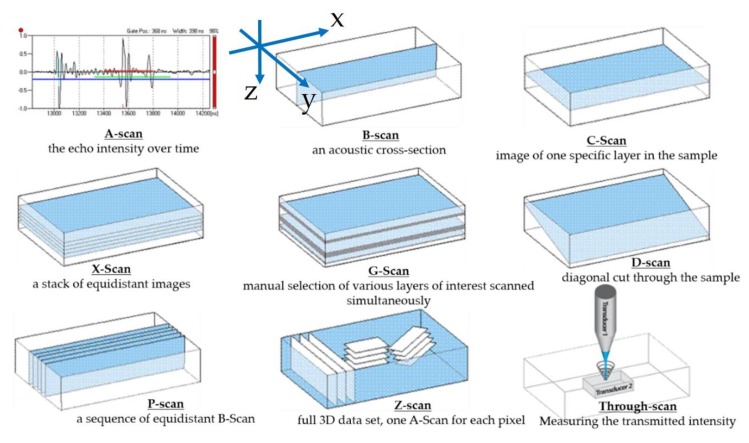
Scan modes of SAM (image courtesy of PVA TePla Analytical Systems).

Different types of analysis modes are available in high definition SAM (Figure 3). The main three modes are A-scans, B-scans, and C-scans. Each one provides different information about the integrity of the sample’s structure. The A-scan is the amplitude of the echo signal over *ToF*. The transducer is mounted on the *z*-axis of the SAM. It can be focused to a specific target layer located in a hard-to-access area by changing the z-position with respect to the sample under testing that is mechanically fixed. The B-scan provides a vertical cross section of the sample with visualization of the depth information. It is a very good feature when it comes to damage detection in the cross section. A C-scan is a commonly used scanning mode, which gives 2D images (slices) of a target layer at a specific depth in the samples; multiple equidistant layers are feasible through the X-scan mode.

## 3. Manufacturing Process of US Probes: The Technical Problem

The growing demand for geometrical precision and the guarantee of high process capability by limiting material damage is a continuous task that engineers must chase. The manufacturing process of miniature ultrasound probes for medical imaging requires homogeneous layers with specific thicknesses (from some microns up to 500 µm) by the bonding between materials with different thermal expansion coefficients and densities. Epoxy adhesive is usually adopted to bond the layers in US probes to each other: The thickness of the bonding line must be as small as possible, ideally less than 1 µm (generally less than 4 µm depending on the acoustic stack structure and its operating frequency), and free from air bubbles. The bonding process variability is strongly related to the surface finishing of the materials, deposition techniques, involved operators, and ambient conditions. Furthermore, multi-element arrays are obtained by means of mechanical dicing. The latter is a critical manufacturing process step, and the fundamental requirement of strong and homogeneous adhesion between materials must be satisfied.

The generation of internal defects, e.g., micro-cracks and delamination, or the realization of non-homogeneous layers of epoxy adhesive is not tolerable. The latter can lead to poor quality or failure of the US probe. SAM seems to be an attractive non-destructive testing for the detection of failure during the manufacturing process of US probes combined with less preparation time for the inspection, reducing the costs of scrapped material.

### Ultrasound Probe for Medical Imaging

**Figure 4:**
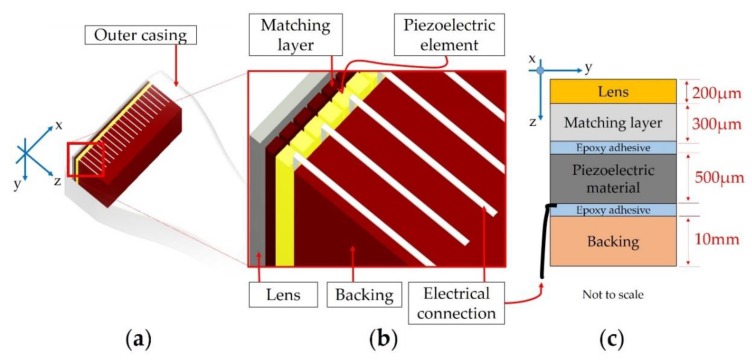
Typical phased array ultrasound probe for medical imaging: (**a**) US probe; (**b**) acoustic stack for an ultrasonic transducer; (**c**) cross section of the US probe (not to scale): the ideal thickness of epoxy adhesive bonding lines (blue color) is less than 1 µm.

The authors considered a US probe, i.e., phased array probe, for medical imaging devoted to cardiac muscle diagnosis that is able to fit between ribs in the intercostal spaces (Figure 4).

The US probe is in direct contact with the patient to detect the dynamic movement of the interior organ under examination and to reveal details of blood flow in real-time. The ultrasound technique is non-invasive, easy to use, low-cost, and painless, and unlike X-ray imaging, there is no ionizing radiation exposure, so it is widely used. The US probe is realized, as shown in Figure 4a, according to the criteria used to design commercially available piezoelectric probes [34].

In particular, the acoustic stack (Figure 4b) is the core of the ultrasound probe. The general structure is based on electrical connections connected to piezoelectric array elements, which are the source for ultrasound pulses. The latter are reflected by the boundaries of the organ structure, in accordance with differences in the acoustic impedance by generating echoes, and they are received by the same acoustic stack acting as an ultrasonic sensor. After the conditioning of the received signals and the processing of all data, the 2D image can be displayed and analyzed on a monitor, in which the doctor can determine the medical diagnosis. The active piezoelectric material, i.e., PZT, is the main component that is divided by means of mechanical dicing, in order to realize an array of elements. The ultrasonic transducer is characterized by 128 piezoelectric elements that are arranged in line 64 on the odd side, and 64 on the even side.

The acoustic matching layer is bonded or casted on the piezoelectric material. The role is to mitigate the potential multiple reflections of the US pulses transmitted by the piezoelectric elements towards the target under testing due to a big difference in the acoustic impedance. Therefore, the matching layer improves and optimizes the US probe sensitivity thanks to the design of adequate acoustic impedance, which is a tradeoff between the high value for the piezoelectric material and the low value of the lens (outer layer of the acoustic structure). The latter is not only in direct contact with the skin of the patient but also focuses the ultrasound beam in the plane perpendicular to the imaging. In the manufacturing process, the bonding of the materials (e.g., the adhesion between the matching layer and piezoelectric material) plays a crucial role in guaranteeing the process capability. The other fundamental component is the backing block, which provides mechanical support for the piezoelectric material and also mitigates acoustic noise effects.

The choice of materials involved in the acoustic stack for an ultrasonic transducer (Figure 4c) with specific electromechanical characteristics is very important for assuring, at the same time, the best image quality and cost reduction for manufacturing.

## 4. Methods

### 4.1. Experimental Setup of SAM

The acoustic stack (Figure 4b) was placed in the water tank of the SAM device (SAM 301 HD^2^—PVA TePla Analytical Systems GmbH, Westhausen, Germany). The sample and SAM transducer were completely plunged in deionized, degassed water to ensure the coupling of the ultrasound waves into the sample. The latter is homogeneous (no air bubbles are entrapped) and it is a non-perishable material. The setup, consisting in the positioning and alignment of the SAM transducer, is very important, in order to guarantee the reproducibility and repeatability of all measurements. A setting is needed for the implemented measurement protocol by considering the multilayered complexity of the US probe acoustic stack under testing. The scanning acoustic microscopy images were obtained by setting a high definition dual gantry system with one channel. The configuration allowed for operation with transducer frequencies up to 400 MHz. An additional tone-burst-module, which extends the operative frequency range up to 2 GHz, was available. The smallest scan resolution for the SAM 301 HD^2^ system is 0.5 µm/px.

The values of the sound speed and thickness of the layers are essential for defining the optimal SAM set-up. Therefore, the SAM transducer frequency was set at 30 MHz (tradeoff between the resolution and penetration depth) and a focal length of 13 mm (chosen as reference), in order to pilot the maximum evidence of defects. The depth of field was 49 µm. The echo-signals of the SAM transducer were digitized by means of an ADC card with 5 GSamples/sec. The software control was WINSAM 8.

**Figure 5:**
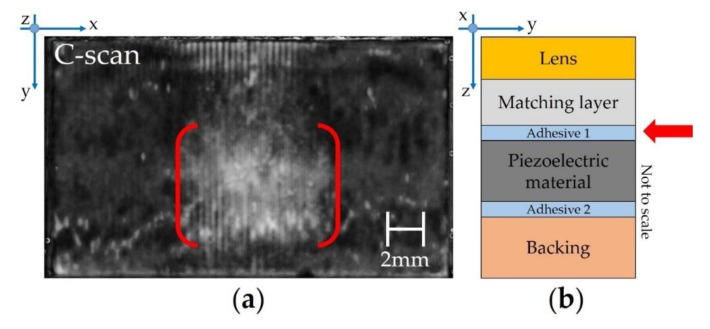
SAM image of adhesive 1 layer: (**a**) C-scan; (**b**) location of the detected layer in the acoustic stack.

The C-scan of the adhesive layer 1 (Figure 5a), which is located between the matching layer and piezoelectric material (Figure 5b), is shown.

The data gate of the C-scan acquisition was set to the *ToF* position corresponding to the depth of the adhesive layer 1 (≈330 ns); the date gate width reflects the thickness of the layer. Following this, a C-scan in the peak-detection mode was recorded with a scan resolution of 20 µm/px. The SAM transducer detected the reflected ultrasound waves every 20 µm, the software evaluated the maximum intensity within the defined data gate (peak detection), and, based on that, a corresponding grey color value was assigned to every pixel. The light grey area in the center of the US probe ultrasonic transducer (inside red colored brackets) shows the significant reflected intensity and reveals potential defects in the internal structure.

### 4.2. Preparation of the Samples

**Figure 6:**
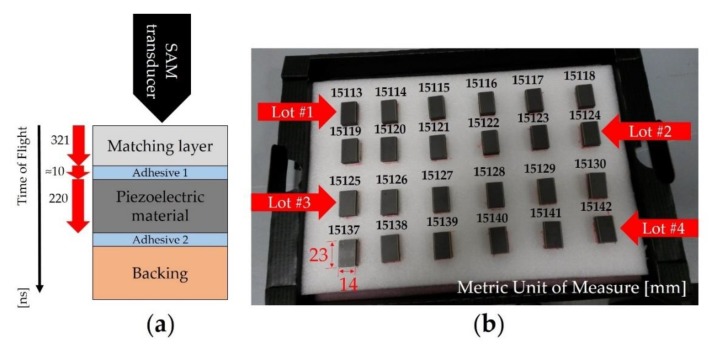
Samples selected for the SAM measurement campaign: (**a**) cross section of the samples under testing (not to scale) with the time of flights of the detected layers; (**b**) 24 acoustic stacks for ultrasonic transducer divided into 4 lots.

We decided to focus on the quality check of acoustic stacks through the inspection of the matching layer, piezoelectric material, and bonding line included between these two layers (Figure 6a). Furthermore, in order to detect potential defects in the middle of the manufacturing process, we did not finalize the ultrasonic transducers into an array of piezoelectric elements by means of mechanical dicing, and with the bonding of the lens (final stage). Due to the preliminary information about the defects (light grey) visualized in the center of the acoustic stack (Figure 5) and located in the adhesive 1 layer, we did not dice the samples by eliminating an additional variability source for the generation of micro-cracks, chipping, or delamination [35,36]. For this reason, we prepared 24 samples by organizing 4 lots, with 6 ultrasonic transducers per lot (Figure 6b). The latter are completely manufactured in the site of Esaote S.p.A. located in Florence (Italy). The production line for this type of acoustic stack is organized in batches, in which different components are assembled through step-by-step processes [37]. The manufacturing process based on a batch size of 6 pieces was principally chosen due to the maximum capacity of the machine (dicing saw) devoted to mechanical dicing for this specific product.

For this study, the bonding process of the samples was realized by a single operator in a controlled environment (24 ± 1 °C) with relative humidity less than 50%. The variability of the bonding process is mainly related to the surface finishing quality of the materials (i.e., roughness of the piezoelectric material, backing, and matching layer). The manufacturing process is composed by a soldering process step between the electrical connections and piezoelectric material (not shown in Figure 6a) and by two sequential bonding steps, the first between the backing and the piezoelectric material and the second between the PZT-based material and the matching layer.

### 4.3. Measurement System for Electroacoustic Characterization of the US Probe Acoustic Stack

**Figure 7:**
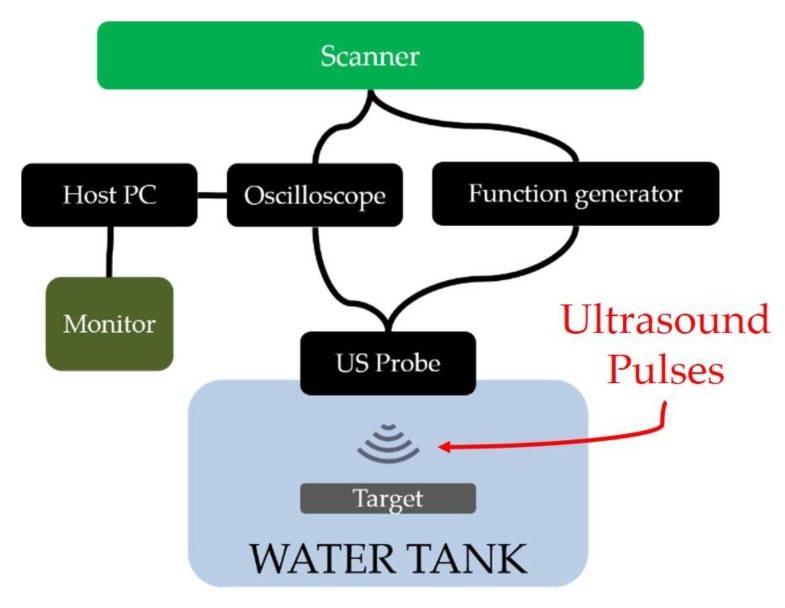
Measurement chain devoted to the pulse echo of the US probe.

In order to assess the final quality of the acoustic stacks, every single piezoelectric element of the array was characterized by means of an ad-hoc measurement system (Figure 7) [38,39]. Three main devices composed the electroacoustic characterization chain: function generator (Agilent 33250A—Santa Clara, CA, USA), digital oscilloscope (LeCroy Waverunner LT342—Chestnut Ridge, NY, USA), and in-house scanner developed and realized by Esaote. The latter is constituted by a multichannel connector, system of a multiplexer/de-multiplexer, and switch, which is able to select one or more elements of the array connected to a general-purpose interface bus to a host computer with a monitor. Once the manufacturing process was completed by bonding the silicone-based lens, the US probe was completely plunged in deionized degassed water and the ultrasound pulses were propagated from the piezoelectric elements to a metallic target. The pulses were totally reflected and the bounces (reflections) were detected as echoes by the US probe [40]. The measurement process started with the alignment of the US probe lens with respect to the target. This step is necessary in order to ensure that all elements were in the maximum reception condition.

The peak amplitude of the echo is a fundamental parameter that allows for characterization of the quality and goodness of the acoustic stack. In fact, the US probe response is defined as follows:

(4) $$Response\;\left[dB\right]=20\cdot \log\left(\frac{V_{RX}}{V_{TX}}\right).$$

**Figure 8:**
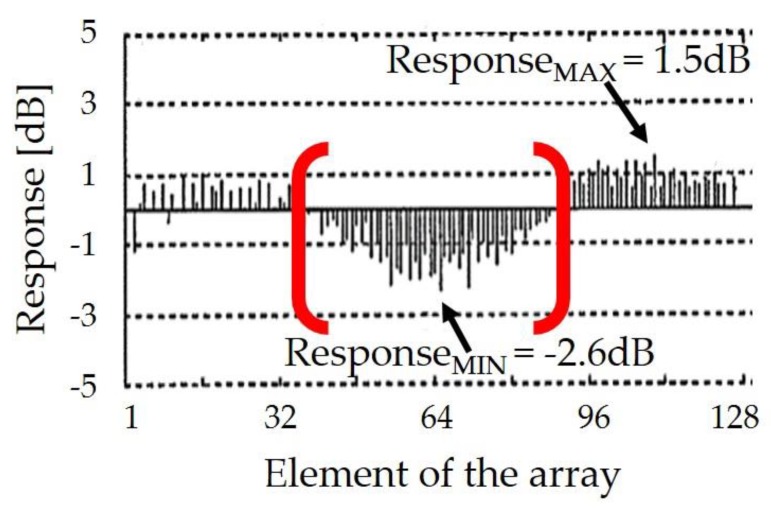
Measurement of the response (electroacoustic characterization) for all 128 array elements.

In Equation (4), *V_RX_* is the peak-to-peak amplitude of the received echo, whereas *V_TX_* is the peak-to-peak amplitude of the transmitted ultrasound pulses. In order to evaluate an important requirement for the probe compliance, i.e., the homogeneity of the responses of the array elements, the electroacoustic characterization based on *Response* is shown in Figure 8. The result is a diagram that represents the *Response* of each element of the piezoelectric array compared to the normalized average value, in decibels. In order to verify the functionality of all the elements, it is possible to select each of them with a mobile marker on the diagram, observing the corresponding echo signal on the digital oscilloscope. For this study, the homogeneity of the acoustic piezoelectric responses was calculated by Equation (5), in which Δ*Response* is the difference between the highest peak-to-peak amplitude response, *Response_MAX_*, and the lowest peak-to-peak response, *Response_MIN_*.

The variation between the maximum and the minimum *Response* by considering the 128 piezoelectric array elements is measured in dB:

(5) $$\Delta Response\;\left[dB\right]=\;Response_{MAX}-Response_{MIN}.$$

To assess the compliance of the acoustic stack quality, we assumed that the direct measure based on Δ*Response* must be in the range 0–4 dB. Over 4 dB, the US probe is considered as a scrap by leading to an additional cost.

The diagram in Figure 8 shows a Δ*Response* greater than the threshold (4.1 dB) and it corresponds to the electroacoustic characterization of the ultrasonic transducers used for the SAM setup (Section 4.1). By considering the *Response* of each piezoelectric element located between the 40th and 90th position, a preliminary correlation can be found between the light grey area (Figure 5a) and the drop of *Response* (from −1 up to −2 dB in the red brackets of Figure 8).

## 5. Experimental Results

We decided to scan lot by lot by means of X-scan analysis (multiple C-scan generating a stack of equidistant 2D-images) by simulating the potential introduction of this NDT method in the middle of the manufacturing process, in order to detect potential defects in the acoustic stacks and to quantify the cycle time of the SAM inspection for six samples. The complete cycle time for scanning a test batch was about 30 min based on the desired image resolution. Finally, the acquired data were processed. The latter were analyzed, and then the acquired data for inspection were processed.

**Figure 9:**
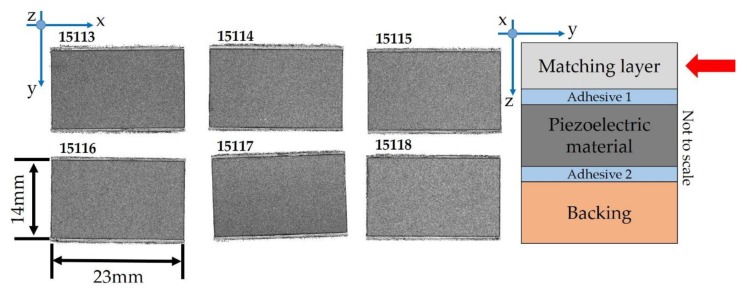
C-scan of the matching layer (lot #1). The surface area is around 322 mm^2^.

As depicted in Figure 9, no defect in the matching layer was found.

**Figure 10:**
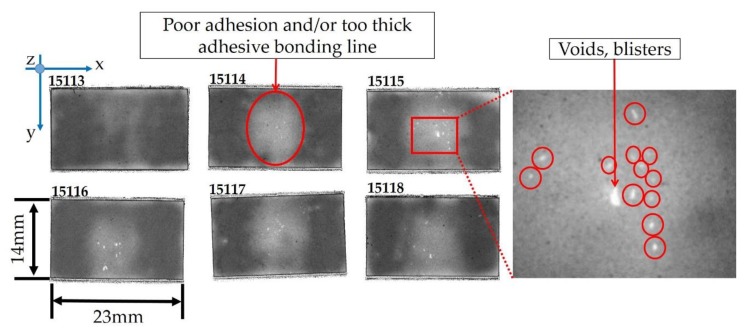
C-scan of the adhesive 1 between the matching layer and piezoelectric material (lot #1).

Defects were detected at the bonding line, i.e., adhesive 1, between the matching layer and the piezoelectric material (Figure 10). By increasing the contrast and reducing the data gate, voids, and blisters (irregular shaped corresponding to the white areas), poor adhesion and/or thicker adhesive bonding line (light grey areas), good adhesion, and/or a layer of epoxy resin with a thickness less than 4 µm (dark grey areas) were shown.

**Figure 11:**
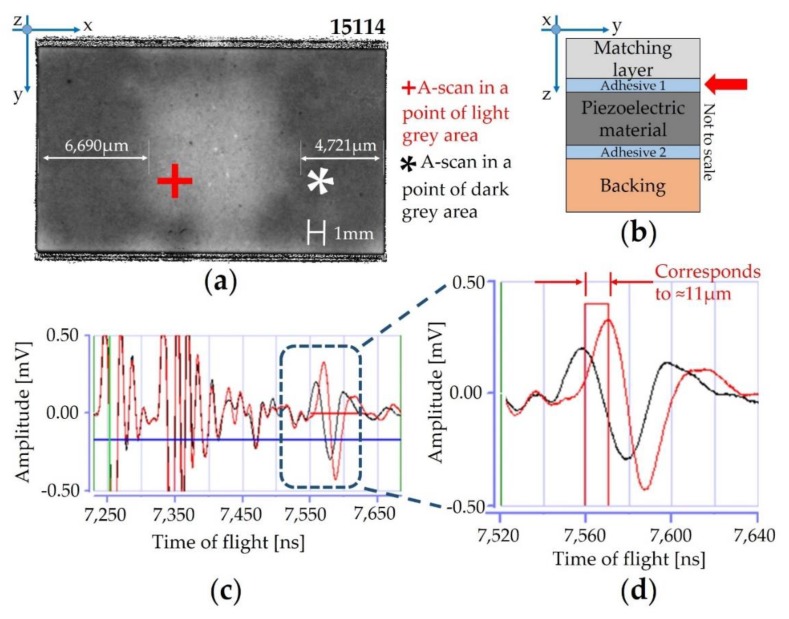
SAM characterization of the sample 15,114—lot #1: (**a**) the two different points with different bonding lines; (**b**) layer under detection; (**c**) A-scans diagram; (**d**) zoom of the A-scans for the adhesive layer 1.

The irregular shape and proximity to each other allowed for the identification of air bubble entrapment recognized as voids (from 10 µm up to 50 µm of dimension) in the central part of the sample (e.g., 15,115), in which poor adhesion or a thicker adhesive bonding line were detected too. In order to analyze in detail the light grey area located in the middle of the acoustic stacks (Figure 11b), we compared the A-scan signals in two different points of the adhesive 1 area (Figure 11a).

In fact, two different A-scans were compared to each other with the purpose of understanding better whether the enhanced reflected intensity at the central part of the areas is caused by poor adhesion or by a thicker epoxy bonding line (Figure 11c): The red wave corresponds to a point in the light area, whereas the black wave corresponds to a point in the dark grey area. The dashed box in Figure 11c denotes the *ToF* position of signals coming from adhesive 1, whereas the red horizontal line corresponds to the data gate that was used to record the C-scan image. The black and red waves are identical before the dashed box (in fact, no defect is recognized in the inner layer, i.e., matching layer), but there is a significant shift in the *ToF*-position for the adhesive layer. The red wave form (Figure 11d) is shifted to greater *ToF* values with respect to the black wave, proving that the light grey area of adhesive layer 1 is thicker than the dark grey area. The thickness difference between the two points was measured around 11 µm (Figure 11d) by considering Equation (3) and using 2000 m/s as the speed of sound of the epoxy resin that makes up adhesive layer 1.

**Figure 12:**
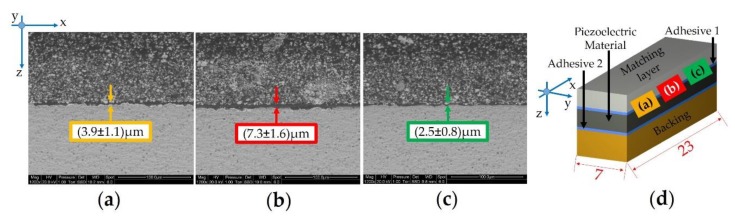
Morphological analysis of the cross section for sample 15,114—lot #1 (not to scale): (**a**) left side; (**b**) central part; (**c**) right side; (**d**) cross section of the acoustic stack.

In order to prove this significant difference in the thickness of adhesive 1, a cross-section of sample 15,114 was prepared as a metallographic specimen before inspection by means of SEM (FEI Quanta 200—Hillsboro, OR, USA) (Figure 12d). The morphological analysis revealed different thicknesses affected by a high standard deviation for the adhesive 1 layer, due to the high roughness of the PZT-based material. By the way, the difference between the thicker adhesive bonding line (Figure 12b—central area) and the peripheral layers (Figure 12a,c) is really significant (3–4) µm. Within the aim of understanding the entity of the non-homogeneous thickness of adhesive 1 layer, advanced signal processing techniques are essential to extract features in detail, which are not easily discernable from measurements. The data post-processing provided by A-scan analysis enhances the capabilities of the SAM method, allowing for measurement of the sample area fraction with thicknesses greater than 4 µm. In this context, the fraction of the scanned area with blisters (voids) was available. After the SAM scans on the overall lots, all the acoustic stacks were completed by executing the expected manufacturing routing to obtain the final US probe’s configuration.

The samples were characterized by means of the measurement system (Figure 7) and the relative Δ*Response* are shown in Table 1.

**Figure 13:**
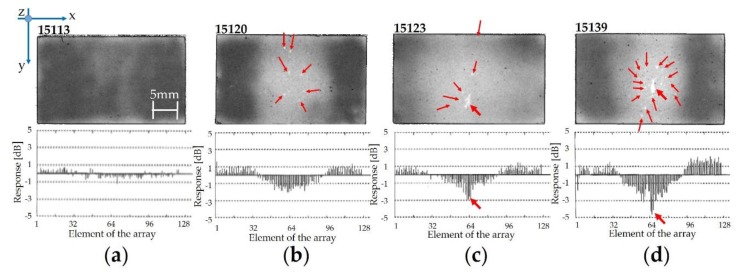
C-scans for adhesive 1 and the corresponding pulse-echo test diagrams: (**a**) sample 15,113 (lot #1); (**b**) sample 15,120 (lot #2); (**c**) sample 15,123 (lot #2); (**d**) sample 15,139 (lot #4).

A very high Δ*Response* (21.8 dB) was measured for sample 15,126, in which the potential root causes can be found in the delamination between the adhesive 1 layer and the matching layer. A comparison between the 2D images by SAM of adhesive 1 with different characteristics and the relative pulse-echo tests is provided in Figure 13.

**Figure 14:**
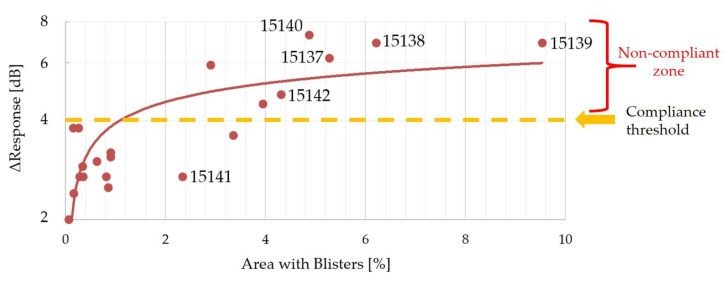
Δ*Response* versus area with voids or blisters.

**Figure 15:**
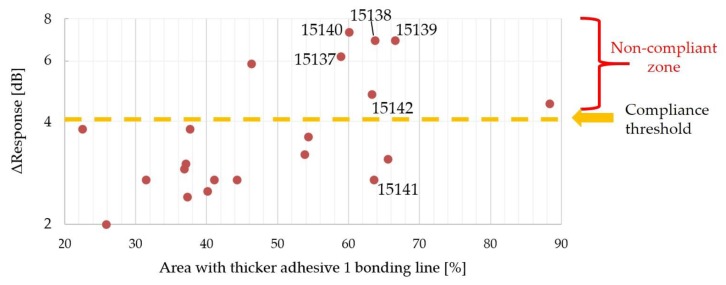
Δ*Response* versus area with thicker adhesive 1 bonding line.

In Figure 14 and in Figure 15, the Δ*Response* versus the area with blisters and the area with a thicker adhesive bonding line are shown.

**Figure 16:**
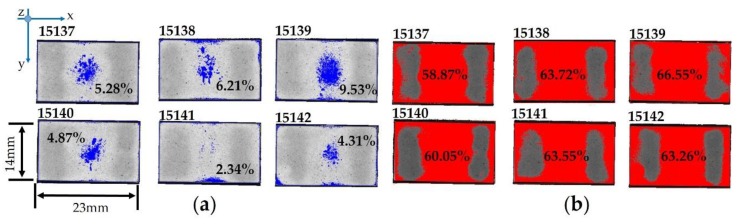
Detection of non-compliant areas [%] for lot #4: (**a**) blisters (blue color); (**b**) thicker epoxy resin (red color).

The two diagrams collect the data for 21 samples, instead of 24 samples, because sample 15,113 was considered as an outlier (scrap) due to the very high Δ*Response* (21.8 dB), 15,114 was analyzed by destructive testing, i.e., SEM, and 15,130 was scrapped for other root causes due to the handling operations. In particular, lot #4 (from 15,137 to 15,142) is affected by some defects, especially in the central part of the epoxy resin layer (Figure 16), in which blisters filled with air (blue color) are not negligible. At the same time, the difference in bonding line (red color) is considerable.

## 6. Discussion

The measurement of the cross section for the thicker bonding line (Figure 12b) did not match the thickness difference of 11 µm estimated by means of SAM, likely due to the uncertain sound velocity adopted for adhesive 1. Furthermore, the *ToF* measurement is affected by the thickness variation of all the layers that comprise the sound path. These reasons explain the difference between the SEM and SAM results. However, SAM scans revealed that the deposition of epoxy resin is not homogeneous, and the thickness of the epoxy resin in the central area of the layer is greater than 4 µm, which is considered the reference.

In Figure 13, the Δ*Response* for the acoustic stacks is considerable depending on the *Responses* of each piezoelectric element, in which samples 15,123 and 15,139 are affected by very low values in the central part of the array. Figure 13a corresponds to the best case thanks to the homogeneity of adhesive 1 (large dark grey area) without blisters, in which Δ*Response* is very low (2.0 dB). Figure 13b shows a not-negligible non-homogeneity for the adhesive 1 layer with a high fraction of the thicker bonding line (around 54%). Despite the presence of some blisters (red arrows), which affect the central area of the sample (3.36%), the Δ*Response* (3.6 dB) complies with the quality requirements (less than 4.0 dB). On the other hand, sample 15,123 (Figure 13c) is affected by the greatest area fraction of the thicker bonding line (88.32%) and the entrapped air bubble fraction is around 4%. This case leads to a high Δ*Response* (4.5 dB). The blister fraction (9.53%) and the thicker adhesive 1 bonding line fraction (66.55%) contribute to a Δ*Response* of 6.9 dB (Figure 13d). This latter represents one of the worst cases and it is not acceptable from the quality point of view.

The crucial requirements for an in-process inspection system can be listed as proper data acquisition, accurate data processing, suitable user interface, flexibility of the operation, and effectiveness of the information on the specimen under testing. Therefore, SAM inspection in the middle of the manufacturing process can predict the poor quality and potential failure of an acoustic stack under construction. The discussion on Figure 14 identifies that some samples with blister fractions less than 2% have a Δ*Response* value that is less than the threshold (4 dB—orange dashed line), which is in compliance with the internal requirements. On other hand, the logarithmic trend (red line) grows more and more for the area with blisters greater than 2%, leading to the poor quality of the acoustic stacks (Δ*Response* over 4 dB). As regards Figure 15, better results are obtained for areas with a thicker epoxy bonding line less than 45% (all ultrasonic transducers are in compliance with the threshold of 4 dB). The Δ*Response* does not show a specific trend, but by considering the area fraction with a thicker bonding line greater than 45%, the number of non-compliant acoustic stacks is seven: These scraps are not sustainable especially when the continuous improvement of the manufacturing process must be developed in a more and more intense competitive scenario by reducing the cost of the poor quality. The simultaneous presence of blisters and a great area fraction of thicker epoxy resin (Figure 16) leads to a non-compliant manufacturing process for the adhesive 1 layer. In this analysis, it is important to underline that sample 15,141 (in compliance with the Δ*Response*) is affected by the very high fraction (63.55%) of the thicker bonding line, but the low fraction (2.34%) of the blisters and their location (close to the edge) will open a large discussion and study about the cross-correlation between defects that affects the acoustic stack.

The failure analysis by means of SAM showed that the issue of US probes’ manufacturing process is primarily due to blisters between the piezoelectric material and matching layer, mostly occurring in the central area of the array. Blisters generate poor quality in the production of US phased array probes, in which the Δ*Response* is greater than 4 dB. The thicker epoxy resin in the central part of the array and the non-homogeneity of the adhesive 1 are evident by SAM analysis: These two factors contribute to a non-compliant value of Δ*Response* and lead to a limited process capability by considering this response.

In this context, the reduction of the area with blisters (less than 2%) and the thicker adhesive 1 bonding line (less than 45%) is essential for improving the US probes manufacturing process. Thanks to the accurate and detailed failure analysis by means of SAM, the root cause of the problem of the manufacturing process was identified and a strategy for the solution can be efficiently driven.

## 7. Conclusions and Future Developments

This work dealt with the detection and the visualization of defects on US probes for medical imaging by means of scanning acoustic microscopy. This non-destructive testing technique, using a transducer with a frequency of 30 MHz and focal length of 13 mm, led to the detection of blisters as defects in the micrometer range with irregular shapes and located close to each other. In particular, the phase inversion, time of flight, and amplitude of the reflected signal are important factors that allowed for discrimination of entrapped air bubbles (voids) with respect to delamination, inclusions, and particles. The SAM technique can measure the fraction of the non-homogeneous thickness of an epoxy adhesive greater than a specific reference (4 µm), located in the bulk of the acoustic stacks under test. The electroacoustic characterizations of the complete US probe structures allowed a comparison of the NDT analyses implemented in the middle of the manufacturing process by means of scanning acoustic microscopy. In fact, the areas of the bonding layer with blisters greater than 2% and a thicker epoxy bonding line greater than 45% led to a poorer quality and failure of an acoustic stack under construction. To this end, the measurements by means of SAM allowed for identification of the root cause in the manufacturing process of US probes as well as an evaluation of the quality of the bonding layer. Therefore, SAM is a robust technique that provides an efficient solution for quick identification and location of defects in multi-layered structures (up to 5 min for each piece under test), enabling efficient failure analysis to predict the integrity of US probes. Manufacturers of ultrasound probes and multilayered structures (i.e., silicon wafers) with thicknesses of some microns will find the SAM technique very useful for the continuous improvement of the process capability and product quality. A drawback can be recognized by the immersion of the device under testing in water, with the consequence that it dries the product immediately after testing to avoid the activation of galvanic corrosion or oxidations. Therefore, SAM is not recommended for non-hermetic packaging in electronics.

Many efforts will be addressed to implement corrective actions, with the priority being the elimination of blister creations during the bonding process between the matching layer and piezoelectric material. Secondly, the epoxy-based adhesive application will be studied, in order to deposit and realize a homogeneous and compliant layer by limiting the thickness up to 1 µm (best case). The lack of non-destructive in-process inspection during the manufacturing process can be overcome by means of this technique.

Future developments will investigate the factors that can affect the blister phenomenon during bonding, i.e., roughness of the piezoelectric material, adhesive type, and surface modification for better bonding under vacuum. The study will be focused on the significant factors, in order to optimize the best solution for increasing the process capability and the quality of the acoustic stacks. By considering the SAM capability (the device allows for a large field of scan of 320 × 320 mm), around 200 samples can be processed, with one providing non-destructive quality control of the ultrasonic transducers with a fast inspection throughput (less than 3 h). Based on these features, SAM becomes a special technique that is very useful for in-line inspection during the manufacturing process of US probes, and also for new product development during the design stage.

## Figures and Tables

**Table 1:** Fraction of areas with blisters and thickness greater than 4 µm for adhesive 1 layer. Not applicable (NA) values refer to samples that were scrapped before the pulse-echo test.

Serial Number	Blisters Fraction (%)	Area with Blisters (mm^2^)	Thicker Adhesive 1 Bonding Line Fraction (%)	Thicker Adhesive 1 Bonding Line Area (mm^2^)	ΔResponse (Db)
15113	0.07	0.21	25.82	74.55	2.0
15114	0.04	0.11	33.14	97.05	NA
15115	0.90	2.60	53.81	157.46	3.2
15116	0.34	0.99	36.83	107.76	2.9
15117	0.16	0.48	37.66	110.19	3.8
15118	0.35	1.03	31.47	92.08	2.7
15119	0.28	0.83	41.06	120.30	2.7
15120	3.36	9.79	54.30	159.09	3.6
15121	0.17	0.51	37.28	109.27	2.4
15122	0.63	1.84	37.04	108.57	3.0
15123	3.95	10.94	88.32	258.86	4.5
15124	2.90	8.45	46.30	135.69	5.9
15125	0.82	2.34	44.25	127.77	2.7
15126	2.18	6.24	47.28	138.44	21.8
15127	0.86	2.47	40.09	115.68	2.5
15128	0.26	0.73	22.49	64.90	3.8
15129	0.90	2.57	65.51	189.04	3.1
15130	0.29	0.84	52.52	151.56	NA
15137	5.28	15.29	58.87	174.16	6.2
15138	6.21	17.98	63.72	188.51	6.9
15139	9.53	27.57	66.55	196.82	6.9
15140	4.87	14.10	60.05	177.59	7.3
15141	2.34	6.78	63.55	187.94	2.7
15142	4.31	12.47	63.26	187.10	4.8
